# Supplementary material for: Modeling glioblastoma heterogeneity as a dynamic network of cell states
Source: Mol Syst Biol. 2021 Sep 16;17(9):e10105. doi: 10.15252/msb.202010105 (PMC8444284; doi:10.15252/msb.202010105)
Supplement: Supplementary file 5 — Source Data for Figure 3 [file MSB-17-e10105-s001.zip › Figure3A_sourcedata/GSEA_3065/nosigdb_state4.GseaPreranked.1622718430994/IVY_IT.html]

Details for gene set IVY\_IT[GSEA]

|  || Dataset | state4 |
| Phenotype | NoPhenotypeAvailable |
| Upregulated in class | na\_pos |
| GeneSet | IVY\_IT |
| Enrichment Score (ES) | 0.67486894 |
| Normalized Enrichment Score (NES) | 2.1638799 |
| Nominal p-value | 0.0 |
| FDR q-value | 2.4285713E-4 |
| FWER p-Value | 0.001 |
Table: GSEA Results Summary

  

Fig 1: Enrichment plot: IVY\_IT      
 Profile of the Running ES Score & Positions of GeneSet Members on the Rank Ordered List

  

| PROBE | GENE SYMBOL | GENE\_TITLE | RANK IN GENE LIST | RANK METRIC SCORE | RUNNING ES | CORE ENRICHMENT || 1 | GFAP |  |  | 0 | 0.995 | 0.1710 | Yes |
| 2 | S100B |  |  | 6 | 0.630 | 0.2784 | Yes |
| 3 | GABBR2 |  |  | 9 | 0.508 | 0.3653 | Yes |
| 4 | BEX1 |  |  | 11 | 0.473 | 0.4464 | Yes |
| 5 | PLEKHB1 |  |  | 82 | 0.262 | 0.4801 | Yes |
| 6 | APLP1 |  |  | 102 | 0.246 | 0.5194 | Yes |
| 7 | CHN1 |  |  | 103 | 0.246 | 0.5617 | Yes |
| 8 | ENHO |  |  | 136 | 0.228 | 0.5957 | Yes |
| 9 | UCHL1 |  |  | 232 | 0.190 | 0.6131 | Yes |
| 10 | NEFL |  |  | 308 | 0.172 | 0.6307 | Yes |
| 11 | PLP1 |  |  | 330 | 0.169 | 0.6564 | Yes |
| 12 | NDRG4 |  |  | 384 | 0.157 | 0.6749 | Yes |
| 13 | TAGLN3 |  |  | 531 | 0.133 | 0.6743 | No |
| 14 | AGT |  |  | 1103 | 0.082 | 0.5968 | No |
| 15 | SLC25A18 |  |  | 1363 | 0.068 | 0.5669 | No |
| 16 | BEX2 |  |  | 1754 | 0.054 | 0.5136 | No |
| 17 | STXBP1 |  |  | 1917 | 0.049 | 0.4960 | No |
| 18 | PRKCZ |  |  | 2055 | 0.045 | 0.4818 | No |
| 19 | KCNJ16 |  |  | 2305 | 0.039 | 0.4486 | No |
| 20 | FBXL16 |  |  | 2963 | 0.025 | 0.3477 | No |
| 21 | KIF1A |  |  | 3468 | 0.013 | 0.2691 | No |
| 22 | FAIM2 |  |  | 3983 | -0.008 | 0.1881 | No |
| 23 | ZNF365 |  |  | 4422 | -0.037 | 0.1243 | No |
| 24 | METTL7B |  |  | 5354 | -0.114 | -0.0054 | No |
| 25 | DNM1 |  |  | 5691 | -0.158 | -0.0320 | No |
| 26 | NRGN |  |  | 5889 | -0.204 | -0.0287 | No |
| 27 | CCK |  |  | 6228 | -0.515 | 0.0056 | No |
Table: GSEA details [plain text format]

  

Fig 2: IVY\_IT: Random ES distribution      
 Gene set null distribution of ES for **IVY\_IT**
